# Fractional Transport of Photons in Deterministic Aperiodic Structures

**DOI:** 10.1038/s41598-017-02170-9

**Published:** 2017-05-23

**Authors:** Luca Dal Negro, Sandeep Inampudi

**Affiliations:** 1 Boston University, Department of Electrical and Computer Engineering and Photonics Center, 8 Saint Mary’s Street, Boston, Massachusetts 02215 United States of America; 2 Boston University, Division of Materials Science and Engineering, 15 Saint Mary’s Street, Brookline, Massachusetts 02446 United States of America; 3 Boston University, Department of Physics, 590 Commonwealth Avenue, Boston, Massachusetts 02215 United States of America; 4 Currently at Northeastern University, Electrical and Computer Engineering Department, 360 Huntington Ave, Boston, Massachusetts 02115 United States of America

## Abstract

The propagation of optical pulses through primary types of deterministic aperiodic structures is numerically studied in time domain using the rigorous transfer matrix method in combination with analytical fractional transport models. We demonstrate tunable anomalous photon transport, including the elusive logarithmic Sinai sub-diffusion in photonic systems for the first time. Our results are in excellent agreement with the scaling theory of transport in aperiodic media with fractal spectra, and additionally demonstrate logarithmic sub-diffusion in the presence of multifractality. Moreover, we establish a fruitful connection between tunable photon diffusion and fractional dynamics, which provides analytical insights into the asymptotic transport regime of optical media with deterministic aperiodic order. The demonstration of tunable sub-diffusion and logarithmic photon transport in deterministic aperiodic structures can open novel and fascinating scenarios for the engineering of wave propagation and light-matter interaction phenomena beyond the conventional diffusive regime.

## Introduction

In 1855, Adolf Fick proposed his laws governing mass transport through diffusive media^1^. In particular, Fick’s second law predicted how the concentration *ϕ*(*x*, *t*) of a diffusing substance changes with space and time. Fick showed that in one spatial dimension *ϕ*(*x*, *t*) obeys the well-known diffusion equation ${\partial }_{t}\varphi (x,t)=D{\partial }_{xx}\varphi (x,t)$, where *D* is the diffusion coefficient. Almost 60 years later, in his theoretical study of the Brownian motion Albert Einstein unveiled the microscopic origin of diffusion by introducing a memoryless (i.e., Markovian) random walk model^2^. It is now well-known that the microscopic dynamics of Markovian random walks obeys a stochastic differential equation which, in the continuum limit of vanishingly small steps, reduces to the Fick’s diffusion equation. Conversely, the fundamental solution (i.e., the Green’s function) for the Cauchy problem of the linear diffusion equation can be interpreted as a Gaussian Probability Density Function (PDF) in space, which evolves in time. All moments of this PDF are finite and its variance, or Mean Square Displacement (MSD), is proportional to the first power of time, i.e., $\langle {x}^{2}(t)\rangle \propto t$, which is the hallmark of a standard diffusion process.

However, after Richardson’s 1926 pioneering work on diffusion in turbulent media^3^, many natural phenomena have been discovered that exhibit anomalous transport characterized by a nonlinear scaling of the MSD according to the power law^4^: $\langle {x}^{2}(t)\rangle \propto {t}^{\beta }$, with *β* a real number in the interval [0, 2]. In particular, anomalous sub-diffusion occurs when *β* < 1 and anomalous super-diffusion when *β* > 1. The extremal cases *β* = 1 and *β* = 2 correspond to standard diffusion and ballistic wave transport, respectively. At the microscopic level, anomalous diffusion processes can be described by generalized Continuous Time Random Walks (CTRWs) that capture non-Markovian correlations between different steps of a walker in non-homogeneous random media^5^. Contrary to standard (i.e., uncorrelated) random walks, CTRW models allow for the possibility of incorporating separate statistical distributions for the waiting times and step sizes of the random walker, including long-tailed non-Gaussian distributions with algebraic decays that yield divergent moments, as in the case of Lévy flights^6^.

Remarkable examples of anomalous transport have been recently discovered in various scientific domains such as turbulent plasmas, viscoelasticity, percolation and transport through fractals and porous media, amorphous solids, biological systems, and even unveiled in the internet traffic^7^. Super-diffusive optical transport has been intensively investigated and artificial media that give rise to super-diffusion of photons, called Lévy glasses, have been recently demonstrated^8^.

In this paper, by numerically investigating optical pulse propagation through dielectric structures, we demonstrate transport regimes that are largely tunable from sub- to super-diffusion in photonic media with long-range aperiodic order. Moreover we demonstrate a novel logarithmic-in-time photon transport regime in deterministic pseudo-random systems with multifractal energy spectra and show that the temporal scaling of the MSD follows the law: $\langle {x}^{2}(t)\rangle \sim {\mathrm{log}}^{\nu }(t)$ with a tunable exponent. Such an intriguing phenomenon was theoretically investigated by Sinai in the context of random walks in non-homogeneous random media^9^ and is referred to as ultra-slow Sinai sub-diffusion, or strong diffusion anomaly. Sinai-type transport has never been reported in deterministic electronic or optical systems and photonic structures that exhibit such an unusual property may provide exciting opportunities for novel optical technologies. In particular, tunable anomalous photon diffusion and logarithmic optical transport in deterministic photonic structures offer novel degrees of freedom to engineer time dynamics and wave dispersion phenomena beyond the conventional framework of diffusion theory.

In order to systematically investigate light transport phenomena in controllable aperiodic environments we focus on one-dimensional (1D) dielectric multilayer structures with fractal and multifractal energy spectra. These structures are rigorously described by well-established transfer matrix and trace map techniques, and have been extensively investigated as model systems to explore the rich physics of aperiodic scattering in electronic, acoustic, and optical media^10, 11^. However, the main conclusions of this paper remain valid for more general photonic systems, such as coupled resonator structures or waveguide arrays, that can be exactly modeled using the transfer matrix technique.

## Anomalous transport and fractional wave propagation in aperiodic media

Understanding the influence of positional correlations and aperiodic order on the nature of optical transport in electronic and photonic structures remains to date a challenging problem of fundamental as well as technological interest. Anomalous transport of electron wave packets through 1D quasi-periodic potentials with fractal energy spectra has been intensively investigated in recent years using the transfer matrix approach^12^, and the connection between the spreading of wave packets and the fractality of energy spectra has been established^13, 14^. In particular, both scaling analysis and numerical simulations have suggested that the exponent *β* for quantum wave packets diffusing through a 1D quasi-periodic sequence of scattering potentials can be written as $\beta =2{D}_{2}^{\mu }/{D}_{2}^{\psi }$, where ${D}_{2}^{\mu }$ and ${D}_{2}^{\psi }$ are the fractal dimensions of the spectrum and of the wave functions, respectively^14^. While many fascinating results have been established in recent years by resorting to extensive numerical simulations of electronic transport in fractal systems, their physical interpretation remains vastly unexplored.

A comprehensive analytical framework that effectively captures the asymptotic transport properties of wave excitations in complex media has only recently been established based on generalized kinetic equations^15^. This powerful approach exploits the recently developed mathematical tools of fractional calculus^16^ that provide the physical underpinning for anomalous transport phenomena in the presence of memory and long-range spatial correlations^15^. In particular, it became clear only recently that at the continuum level, CTRWs produce *fractional transport equations* with space and time derivatives of fractional order^17^. These are integro-differential operators with power-law kernels that account for space correlations and time memory effects invariably established when wave excitations are multiply scattered in strongly non-homogeneous environments^16^.

Due to the well-known isomorphism between the Schrödinger and Helmholtz equations in 1D scattering potentials, classical wave scattering shares fundamental analogies with its electronic counterpart. Striking examples are the formation of photonic band gaps in periodic scattering media^18, 19^, the optical Hall effect^20^, optical negative temperature coefficient resistance^21^, and Anderson localization of light waves in disordered random media^22, 23^.

Building on this powerful analogy we use full-vector electromagnetic modeling to demonstrate novel transport phenomena in photonic systems with deterministic aperiodic order beyond what has been established in their electronic counterparts. In particular, we found that the photonic transport can be switched from super- to sub-diffusion by modulating the refractive index contrast and Sinai-type logarithmic sub-diffusion of optical waves can be achieved in deterministic multifractal environments. The nature of optical transport in our paper is investigated by considering the time scaling of the MSD and the temporal autocorrelation function (ACF) of optical wave packets that propagate across photonic multilayers with periodic, quasi-periodic, and pseudo-random positional order.

Aperiodic structures can be rigorously classified according to the nature of their diffraction and energy spectra, which correspond to mathematical spectral measures. According to the Lebesgue’s decomposition theorem, any measure *μ* can be uniquely decomposed in terms of three primitive spectral components (or mixtures of them), namely: pure-point (*μ*_*P*_), singular continuous (*μ*_*SC*_), and absolutely continuous spectra (*μ*_*AC*_), such that *μ* = *μ*_*P*_ + *μ*_*SC*_ + *μ*_*AC*_. The structures investigated in this work are the chief representatives of each spectral class. Fibonacci structures are quasi-periodic and their diffraction spectra are pure-point, featuring a countable set of *δ*-like Bragg peaks at incommensurate intervals. On the other hand, their energy spectra are singular continuous and converge to a Cantor-set. More complex structures exist, such as the Thue-Morse ones^24^, which display singular continuous diffraction and fractal energy spectra. The individual Bragg peaks in such systems are not separated by well-defined intervals and form broad bands in Fourier space. These systems are structurally more complex than quasi-periodic ones. Interestingly, their optical properties have been found to be closer to the ones of periodic structures^25^. Finally, Rudin-Shapiro structures^10^ are pseudo-random with absolutely continuous diffraction spectra and pure-point energy spectra akin to random media in the localization regime. However, the nature of their eigenmodes and their localization properties are not yet fully understood^26^. For instance, differently from fractal structures such as Fibonacci and Thue-Morse ones, the integrated density of states of Rudin-Shapiro structures was found to scale logarithmically and their energy spectra display multifractal behavior for certain values of the scattering strength^27^. In this paper we demonstrate that such unusual spectral properties give rise to logarithmic transport of optical waves in such systems for the first time.

**Figure 1:**
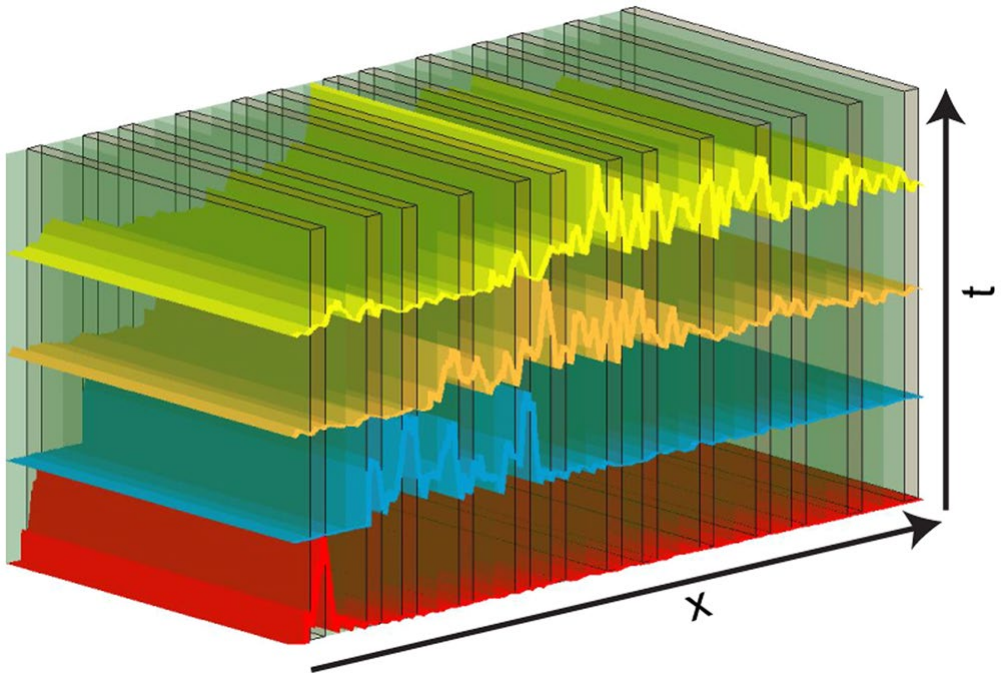
Schematic of a set of scattering layers with representative fractal pulse shapes displayed at different times.

The device structures considered in this work are all generated by binary inflation rules that act on two constituent layers (say A and B) with refractive indices *n*_1_ and *n*_2_, respectively, as schematically represented in Fig. 1. With no loss of generality the optical thickness of each layer is set to be λ_0_/4, and we chose λ_0_ = 1550 *nm*. The spatio-temporal electric field distribution of propagating pulses and the optical transmission spectra are calculated using the rigorous transfer matrix method over a wide frequency range centered at ${\omega }_{0}=2\pi c/{\lambda }_{0}$. Additional details on this well-established technique can be found in our methods section. In analogy with the electronic case, we define the MSD of optical wave packets as^14, 28^:

1 $$\langle {x}^{2}(t)\rangle =\frac{1}{L}{\int }_{0}^{L}{|x-{x}_{0}|}^{2}{|E(x,t)|}^{2}dx=\frac{1}{L}{\int }_{0}^{L}{|x-{x}_{0}|}^{2}{|{\int }_{0}^{\infty }{E}_{i}(\mathrm{0,}\omega )\psi (x,\omega ){e}^{-i\omega t}d\omega |}^{2}dx$$

where *L* represents the total length of the sample, $E(x,t)$ is the electric field inside the sample as a function of space and time (methods section), ${E}_{i}(\mathrm{0,}\,\omega )$ is the frequency spectrum of incident field, and *ψ*(*x*, *ω*) is the scattering map of the system (methods section). Moreover, we study the temporal ACF of optical wave packets defined as^13^:

2 $$C(t)=\frac{1}{t}{\int }_{0}^{t}{|\langle E(x,t^{\prime} )|E(x,\mathrm{0)}\rangle |}^{2}dt^{\prime} $$

where <, > indicates the inner product $\langle E(x,t)|E(x,\,\mathrm{0)}\rangle ={\sum }_{i}E({x}_{i},t)E({x}_{i},\,0)$.

The temporal scaling of the above quantities fully reveals the anomalous nature of diffusive transport in arbitrary 1D scattering systems. In particular, it is well-known that the transport of electronic excitations across quasi-periodic structures with fractal energy spectra displays an asymptotic scaling of *C*(*t*) that decays algebraically as ~*t* ^−*δ*^, where the exponent *δ* is equal to the fractal dimension of the spectrum^13, 14^. Interestingly, this behavior has not been reported for optical wave excitations, motivating our study.

## Transport across localized photon states in periodic structures

**Figure 2:**
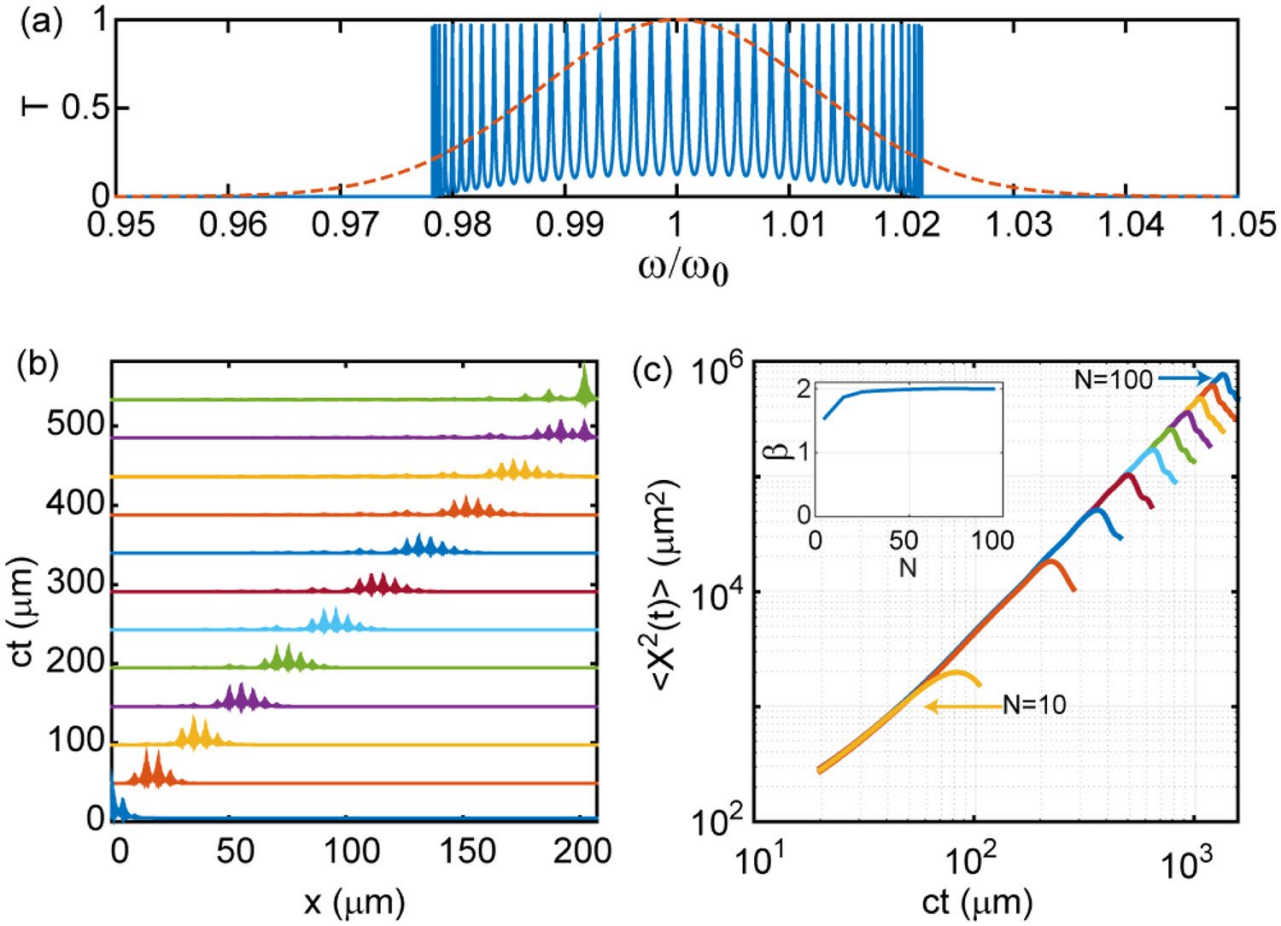
(**a**) Transmission spectrum of *N* = 40 cavities of ${\lambda }_{0}\mathrm{/2}{n}_{1}$ thickness each *λ*_0_ = 1550 *nm*. Cavities are separated from each other by periodic Bragg reflectors made of 20 alternating layers of refractive indices *n*_1_ = 1.5, *n*_2_ = 1.75 and thickness *λ*_0_/4*n*_1_ and *λ*_0_/4*n*_2_, respectively. Dashed line represents the frequency spectrum of the incident optical pulse. (**b**) Intensity distribution $({|E|}^{2})$ of an optical pulse propagating through the layers at different times normalized to the incident pulse. (**c**) Calculated MSD of the pulse in coupled cavity systems with varying cavities ranging from N = 10 to N = 100 (in steps of 10). (Inset) Calculated scaling exponent of the MSD as a function of N. ‘ct’ in (**b**) and (**c**) is the time equivalent length where c represents the velocity of light and t represents time.

In order to validate our computational method we first considered as a reference case the optical pulse transport through localized states in photonic coupled micro-cavities with structural defects distributed periodically along an otherwise regular photonic crystal structure. The defects consisted in additional layers of type A positioned along the periodic AB layer sequence. The investigated system can be symbolically represented as ${[{(AB)}_{M}A]}_{N}{(BA)}_{M}$, where *M* and *N* indicates the number of repeated units inside the parenthesis. This photonic system supports a number of resonant defect modes forming a comb-like structure within its fundamental Bragg gap. The number of localized states in the gap equals the number *N* of cavity defects introduced in the system, which are regularly spaced with respect to the central frequency *ω*_0_ as shown by the calculated transmission spectrum in Fig. 2(a), overlapped with the spectrum of the incident pulse. The transmission spectrum contains a large number of regularly separated resonant states with very close spacings. However, even for large systems with several hundred defect layers, the resonant states always overlap resulting in coherent photon tunneling (i.e., resonant tunneling) across the entire structure. This phenomenon is analogous to the well-known formation of transmission mini bands for the electron transport in semiconductor superlattices. Moreover, despite the large number of resonant modes, this spectrum does not support a fractal structure or fractal eigenstates, and therefore ${D}_{2}^{\mu }/{D}_{2}^{\psi }=1$. According to the scaling theory of electron transport in 1D, no anomalous diffusion is expected to take place under such condition and in fact we show that wave transport occurs ballistically. This qualitative picture is confirmed by our numerical simulations obtained by computing the spatial-temporal evolution of a Gaussian optical pulse transported across the structure (details in methods section). In Fig. 2(b) we show few representative snapshots of the pulse intensity that propagates inside the structure.

The normally incident input pulse tunnels into the system and propagates ballistically while spreading in time until it reaches the output face of the structure. At any spatial position in the sample, the envelope of the pulse remains Gaussian with a high-frequency spatial modulation that results from the interference inside the structure^29^. Calculated MSD for pulses transported across coupled cavities with different numbers of defect states are displayed in Fig. 2(c). The MSD time traces feature a power-law scaling with a constant slope for systems with up to 100 coupled cavities, irrespective of the refractive index contrast in the structure. This behavior demonstrates coherent transport of the wave packet with an asymptotic scaling exponent *β* = 2, which indeed corresponds to the ballistic regime. The inset in Fig. 2(c) summarizes the results obtained for different numbers *N* of cavity (defect) states. Notice that the values of the transport exponent converge to the ballistic value *β* = 2 when the structures are large enough to avoid spurious reflections from their end facets, which cause an artificial drop in the MSD curves. In all our simulations we exercised particular care to verify that the coupling of the pulse to the end facet of the structures is negligible, so that the values of the transport exponents (obtained by power-law fitting) do not depend on the launching conditions of the pulses or on the total length of the system. The ballistic nature of pulse transport in periodic coupled cavity systems is further confirmed by the study of the corresponding ACF time decay, which was found to follow the power law $C(t)\sim {t}^{-\delta }$ with *δ* = 1, irrespective of the index contrast.

## Tunable anomalous photon transport in Fibonacci and Thue-Morse structures

We can now address the optical pulse transport in quasi-periodic Fibonacci and in deterministic aperiodic Thue-Morse and Rudin-Shapiro photonic media. In contrast to periodic structures, these photonic systems exhibit largely tunable anomalous transport as a function of the refractive index contrast between the constituent layers *A* and *B*.

**Figure 3:**
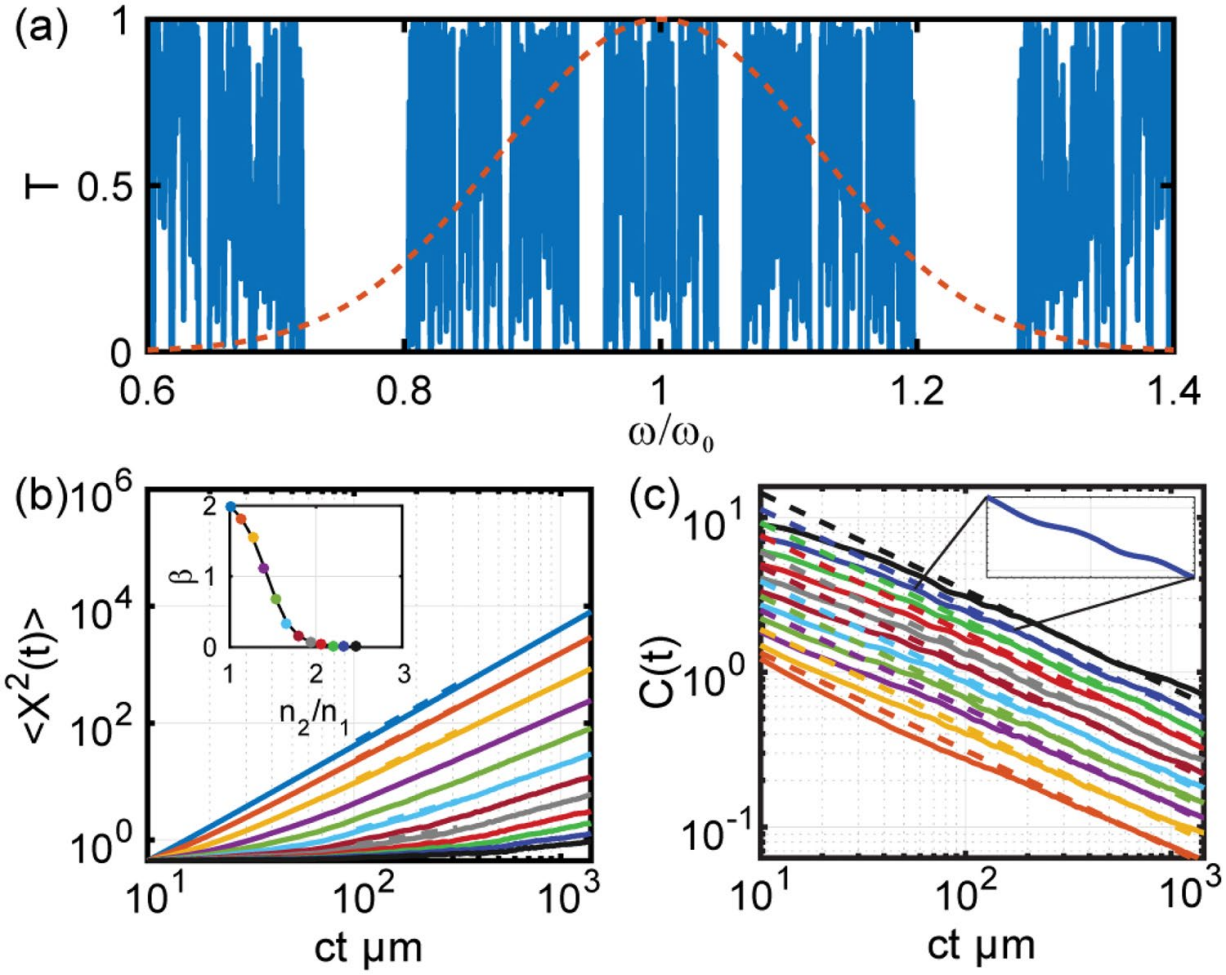
(**a**) Representative transmission spectrum of a Fibonacci multilayer with refractive indices *n*_1_ = 1.5 and *n*_2_ = 1.9 with layer thickness *λ*_0_/4*n*_1_ and *λ*_0_/4*n*_2_ respectively. Dashed line represents the frequency spectrum of the incident optical pulse with central wavelength at 1550 nm. (**b**) Computed MSD values as a function of time and refractive index contrast between the constituent layers (*n*_1_ = 1.5 in all cases), Inset shows the scaling exponent of the MSD obtained using numerical fitting of the data to power law. The quality of the fitting is demonstrated with representative dashed lines overlapping the data in main panel. (**c**) Computed ACF as function of time. Solid lines represent the numerical data and dashed lines are theoretical prediction (Lines are scaled vertically for better visualization). The inset shows a magnified portion of the initial-time ACF decay curve that support fractal log-periodic oscillations. ‘ct’ in (**b**) and (**c**) is the time equivalent length where c represents the velocity of light in vacuum and t represents time.

As a first case we consider a quasi-periodic Fibonacci photonic system where the scattering layers are arranged according to the well-known Fibonacci inflation rule^10, 11^: *A* → *AB* and *B* → *A* starting with *A*. The optical transmission spectrum of this structure is truncated at *N* = 2048 layers, and it is shown in Fig. 3(a) overlapped with the spectrum of the input pulse. It is known that in the limit of *N* → ∞ (where *N* is the number of layers) the highly fragmented spectrum of the Fibonacci multilayer converges to a self-similar Cantor set with zero Lebesgue measure and with fractal dimension *d*_*f*_ = ln2/ln3, irrespective of the refractive index contrast^30^. Moreover, differently from periodic systems, Fibonacci multilayers support distinctive modes, known as *critical states*, with an envelope that decays according to a power law and with a highly fluctuating character described by a distribution of fractal exponents (multifractal states) that vary with frequency and with the strength of the scattering potential^30^. As we will demonstrate in this paper, these distinctive spectral and mode properties provide vastly unexplored opportunities to tailor the optical transport in aperiodic systems.

We first note that exact fractality of the spectrum (at all frequencies) only occurs in the limit of infinite layers. However, almost perfect self-similarity across broad spectral regions can be obtained even for relatively short Fibonacci structures with only few hundred layers. For our study we selected a number of layers that gives rise to a constant fractal dimension across the part of the frequency spectrum that overlaps with the envelope of the propagating pulse. Under these conditions, we show that due to the fractality of the Fibonacci spectrum and the broad distribution of fractal dimensions of the critical eigenmodes^31^ it is possible to achieve anomalous transport with the exponent *β* controlled by the refractive index contrast. To demonstrate this behavior we perform numerical simulations of the MSD scaling, shown in Fig. 3(b) for different values of the refractive index contrast. We found that the slopes of the MSD curves, plotted on a double logarithmic scale, strongly depend on the choice of the refractive index contrast. The strong dependence of the transport exponents *β* on the values of the refractive index contrast is summarized in the inset of Fig. 3(b). Our data clearly demonstrate that the transport properties of optical Fibonacci systems can be tailored from super-diffusion (*β* > 1) to sub-diffusion (*β* < 1) depending on the strength of the scattering potential (i.e., refractive index contrast), in close analogy with the tunable quantum dynamics of quasi-periodic electronic systems qualitatively explained by renormalization group arguments^12^. It should be emphasized that the observed tunability of photon transport is not associated with the excitation of a single critical mode but rather with a distribution of multifractal states that overlap the spectrum of the incident pulse.

**Figure 4:**
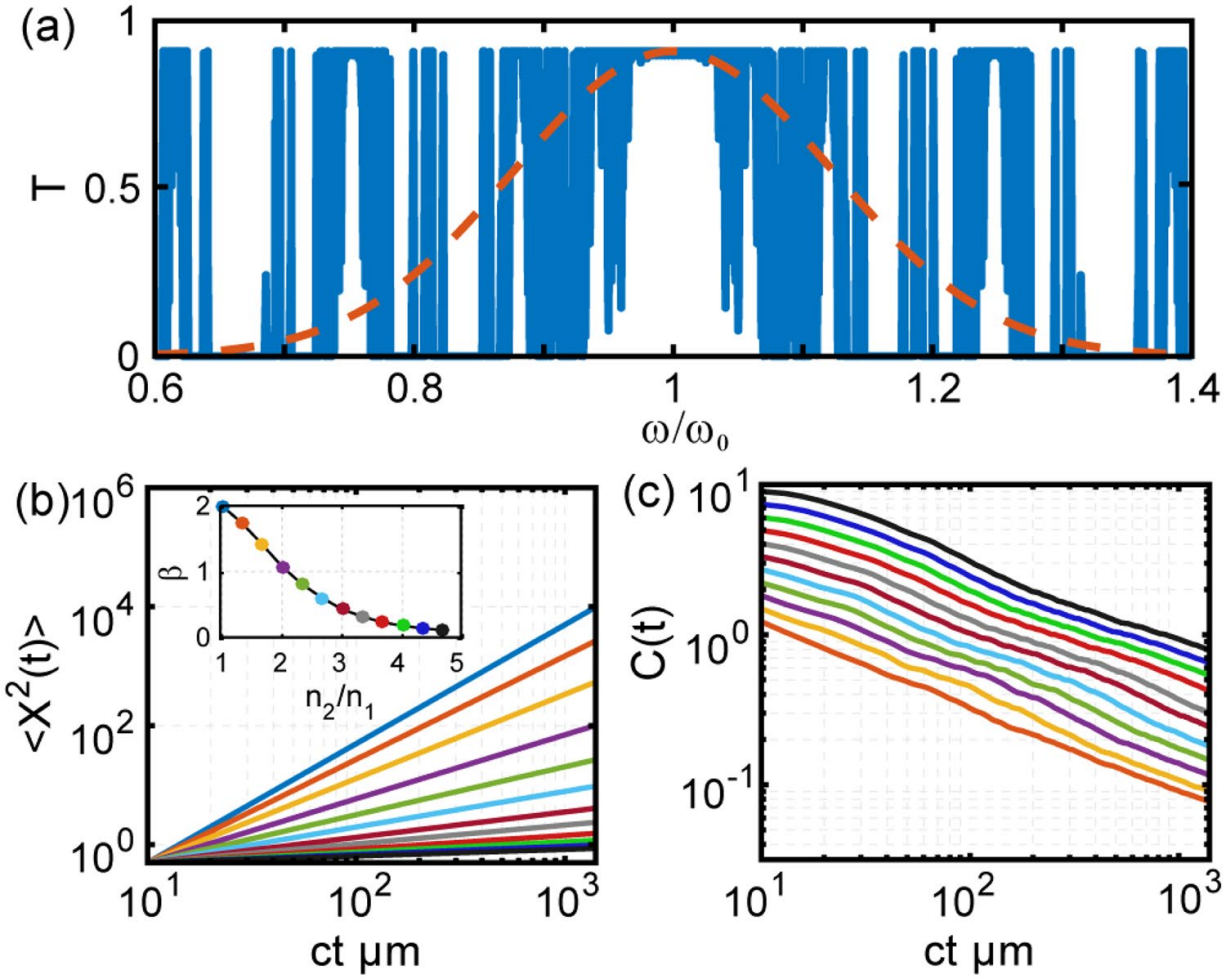
(**a**) Representative transmission spectrum of a Thue-Morse multilayer with refractive indices *n*_1_ = 1.5 and *n*_2_ = 1.9 with layer thickness *λ*_0_/4*n*_1_ and *λ*_0_/4*n*_2_ respectively. Dashed line represents the frequency spectrum of the incident optical pulse with central wavelength at 1550 nm. (**b**) Computed MSD values as a function of time and refractive index contrast between the constituent layers (*n*_1_ = 1.5 in all cases), Inset shows the scaling exponent of the MSD obtained using numerical fitting of the data to power law. The quality of the fitting is demonstrated with representative dashed lines overlapping the data in main panel. (**c**) Computed ACF as function of time. Inset shows the scaling exponent of the ACF obtained using numerical fitting of the data to power law. ‘ct’ in (**b**) and (**c**) is the time equivalent length where c represents the velocity of light in vacuum and t represents time.

Moreover, we discovered a very similar behavior also in Thue-Morse structures^10, 11, 25^ which are generated by the inflation rule: *A* → *AB* and *B* → *BA*. Similarly to the Fibonacci case, these structures support a singular-continuous energy spectrum with self-similar fractal properties^24, 25, 32^. Earlier work has compared the transport properties of Fibonacci and Thue-Morse structures within the tight-binding model and reported anomalous super-diffusion for electrons^33^. Moreover, it was shown that, for a given scattering strength, electrons in Thue-Morse structures are less localized compared to Fibonacci ones and the degree of aperiodicity of Thue-Morse structures is intermediate between periodic and quasi-periodic systems. Our results on the photon transport across Thue-Morse structures with varying refractive index contrast are summarized in Fig. 4. Similarly to the Fibonacci case, we observe widely tunable anomalous transport behavior across the self-similar Thue-Morse structure and clearly demonstrate both super-diffusion and sub-diffusion depending on the refractive index contrast. Moreover, by comparing the decay of the transport exponents shown in the insets of Figs 3 (b) and 4(b) we realize that the transition into the localization regime (*β* = 0) occurs more gradually in Thue-Morse compared to Fibonacci structures. Similarly to the case of electronic transport, this behavior is consistent with the more extended character of the optical modes in Thue-Morse structures. The inset in Fig. 4(c) displays the scaling exponents *δ* of the ACF of the Thue-Morse structure for different values of the refractive index contrast.

## Connection with fractional transport

The anomalous transport behavior discovered in the Fibonacci and Thue-Morse structures can be regarded as an instance of fractional photon transport. The large tunability of photon transport in deterministic aperiodic systems is effectively described by the asymptotic solutions of the *fractional diffusion-wave equation* (FDWE): ${D}_{\ast }^{\beta }\varphi (x,t)=D{\partial }_{xx}\varphi (x,t)$ with $0 < \beta \le 2$. In this equation *D* stands for a generalized diffusion coefficient while ${D}_{\ast }^{\beta }$ is the Caputo-type fractional time-derivative of (real) order *β*, which is defined as^34^:

3 $${D}_{\ast }^{\beta }\varphi (x,t)=\{\begin{array}{ll}\frac{1}{{\rm{\Gamma }}\mathrm{(1}-\beta )}{\int }_{0}^{t}[\frac{\partial }{\partial \tau }\varphi (x,t)]\frac{d\tau }{{(t-\tau )}^{\beta }} & {\rm{if}}\,0 < \beta  < 1\\ \frac{1}{{\rm{\Gamma }}\mathrm{(2}-\beta )}{\int }_{0}^{t}[\frac{{\partial }^{2}}{\partial {\tau }^{2}}\varphi (x,t)]\frac{d\tau }{{(t-\tau )}^{\beta -1}} & {\rm{if}}\,1 < \beta  < 2\end{array}$$

and Γ is the Euler’s gamma function. When *β* is an integer (*β* = 1, 2) the right-hand-side in the above definition reduces to the corresponding partial derivative of integer order and we recover either the standard diffusion equation for *β* = 1 or the wave equation for *β* = 2. When 1 < *β* < 2 the fractional equation is expected to interpolate continuously between a diffusion and wave propagation. The large tunability of wave transport associated to the fractional order *β* manifests the microscopic non-Markovian nature of photon transport in complex aperiodic systems. In fact, the FDWE kinetics provides an effective model to account for the complex photon correlations and memory effects established by the phases of multiply scattered waves in strongly inhomogeneous aperiodic media. The connection between field propagation through multilayered structures with fractal spectra and fractional transport is established based on the scattering matrix approach^35^ in our methods section. In particular, it is possible to show based on simple scaling arguments that the time-dynamics of optical pulses in fractal scattering systems is determined by a non-local fractal operator in time-domain^15, 36^ that depends, yet in a complex and non-analytical fashion, on the aperiodic refractive index modulation.

The fundamental solution of the FDWE can be obtained in closed-form using Fourier-Laplace integral inversion methods (i.e., Mellin-Barnes integrals) and it can be expressed analytically in terms of the transcendental Wright functions or using the Fox H-functions^34, 37^. The reduced Green’s function (see methods section), can be interpreted as a symmetric spatial PDF evolving in time with a stretched exponential relaxation, which provides the following expression for the MSD^37^:

4 $${\sigma }^{2}=\frac{2{t}^{\beta }}{{\rm{\Gamma }}(\beta +\mathrm{1)}}$$

It is essential to notice here that the transport process predicted by the FDWE model is widely tunable and can be switched from anomalous sub-diffusion (0 < *β* < 1) to anomalous super-diffusion (1 < *β* < 2).

As we appreciate in Figs 3 and 4, our numerical results on the MSD scaling can be accurately described by considering the asymptotic solutions of the FDWE model, which are shown by the dashed lines. A similar FDWE approach has been recently proposed to model the multiple scattering of acoustic waves in one-dimensional multiscale media with long-range spatial correlations^38^. In this context, the FDWE is associated to lossless inhomogeneous random media and describes an effective medium with power-law frequency dependent attenuation coefficient^38^. The fractional wave transport approach is well-established in fields such as viscoelasticity and seismic wave propagation^39^, but it has yet to receive proper consideration in optics. However, from a physical standpoint fractional transport naturally follows from the fractal memory kernel in the field equation governing the dynamics of optical waves in aperiodic media with self-similar spectra. A heuristic scaling analysis of the MSD and ACF scaling of photonic systems with fractal spectra is described in our methods section.

The fractal nature of the Fibonacci and Thue-Morse transport is directly revealed by the ACF decay, displayed in Figs 3 (c) and 4(c) for varying refractive index contrast. All the ACF curves exhibit an inverse power law scaling with constant slope. The decay exponent *δ* is found to be independent on the refractive index contrast, in agreement with the analytical scaling law $C(t)\sim {t}^{-\delta }$ previously introduced for electronic systems. We further validated our numerical results by computing the fractal dimension of the Fibonacci transmission spectrum using the accurate Wavelet Transform Modulus Maxima (WTMM) technique^40–42^ as summarized in the methods section. This analysis confirms that the calculated fractal dimension (*d*_*f*_ = ln2/ln3) of the Fibonacci spectrum equals the exponent *δ* independently estimated from the power-law fitting of the ACF decay. Finally we show in the inset of Fig. 3(c) a magnified portion of a typical ACF decay curve to emphasize the presence of initial-time log-periodic oscillations that develop in close analogy to the case of electronic systems^12^. Such oscillations have been recently addressed using the analytical theory of spectral zeta functions on fractals^43, 44^. Physically, they manifest resonant scattering phenomena between neighboring lattice clusters that share similar local geometrical structures in fractal environments. This intriguing phenomenon was also discussed in the context of the electron transport across Fibonacci chains using the renormalization group approach^45^. Interestingly, we report similar oscillations in Thue-Morse structures for the first time.

## Sinai logarithmic photon transport in Rudin-Shapiro structures

**Figure 5:**
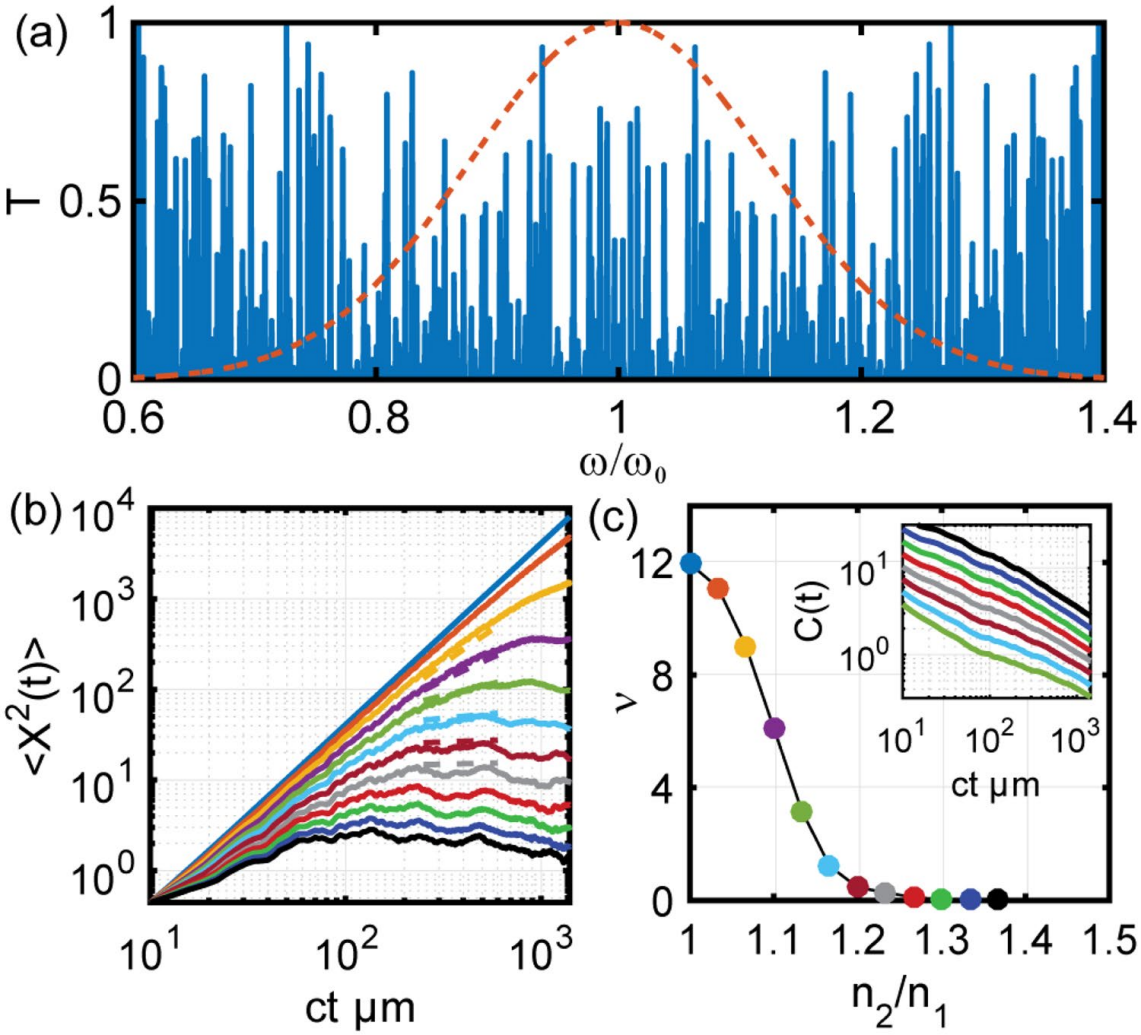
(**a**) Representative transmission spectrum of an RS multilayer with *n*_1_ = 1.5 and *n*_2_ = 1.75. The dashed line represents the frequency spectrum of the incident optical pulse at 1550 nm. (**b**) Calculated MSD scaling curves for different values of the refractive index contrast between the constituent layers. MSD scaling is observed to have logarithmic behavior in a double-log plot representing ultra-slow Sinai diffusion. The deviation from logarithmic behavior at higher time scales is due to the finite size of the system of layers (reflections from the other end of the layers). (**c**) Calculated scaling exponent of the MSD curves shown in panel (**b**) using the logarithmic Sinai model. The quality of the fitting is demonstrated with representative dashed lines overlapping the data in panel (**b**). Inset in (**c**) is calculated ACF for few representative refractive index contrasts. The lines are scaled vertically for better visualization. ‘ct’ in (**b**) and (**c**) is the time equivalent length where c represents the velocity of light in vacuum and t represents time.

We now address Sinai logarithmic sub-diffusion of optical wave packets in Rudin-Shapiro (RS) photonic structures. RS structures features unique spatial correlations associated to an unusual scaling of the density of states that can be described by multifractal analysis^26^. Indeed, in our method section we utilize the WTMM analysis to compute the multifractal spectrum of the RS optical transmission. RS multilayers are generated from a two-letter alphabet subject to the simple inflation rule: *AA* → *AAAB*, *BB* → *BBBA*, *AB* → *AABA*, *BA* → *BBAB*, starting from the initial seed *AA* ^11^. The transmission spectrum of the RS photonic structure (N = 2048 layers) shown in Fig. 5(a) is characterized by a singular distribution of narrow resonant peaks. Figure 5(b) summarizes our results for the MSD of optical wave packets propagating through the systems. The data are plotted in a double logarithmic scale and demonstrate a clear logarithmic scaling behavior, in stark contrast to the case of Fibonacci and Thue-Morse systems. To the best of our knowledge this behavior is not displayed by other deterministic optical systems and provides exciting opportunities to engineer ultra-slow diffusion phenomena using conventional dielectric structures.

Logarithmic sub-diffusion also falls within the very general umbrella of fractional kinetics. In fact, at the continuum level, Sinai logarithmic sub-diffusion is described by a fractional diffusion equations of distributed-order^46, 47^. The distributed-order time fractional diffusion equation can be obtained from the FDWE by integrating with respect to the order *β* the fractional time derivative as follows:

5 $${\int }_{0}^{1}p(\beta ){D}_{\ast }^{\beta }\varphi (x,t)d\beta =D{\partial }_{xx}\varphi (x,t)$$

where $p(\beta )$ is a non-negative dimensionless weight function subject to the normalization condition ${\int }_{0}^{1}p(\beta )d\beta =1$. Clearly when the weight is a delta function the distributed-order time fractional diffusion equation reduces to the FDWE as a special case. Ultra-slow kinetic processes with tunable logarithmic MSD scaling $\langle {x}^{2}\rangle \sim {\mathrm{log}}^{\nu }(t)$ manifest the asymptotic solution of the distributed-order diffusion equation with a power-law weight function $p(\beta )=\nu {\beta }^{\nu -1}$ (*v* > 0) that is associated to a very broad distribution of localization traps (i.e., localized modes). Therefore, distributed-order diffusion equations result from microscopic CTRWs processes with extremely broad waiting time PDFs, characteristic of strongly non-homogeneous environments. The case *v* = 4 corresponds to the original Sinai model^46^.

The dashed lines in Fig. 5(b) are best fits obtained according to the general Sinai model $\langle {x}^{2}\rangle \sim {\mathrm{log}}^{\nu }(t)$ ^46^. Notice that also in the case of the RS structure distinctive oscillations develop in the MSD curves, reflecting the correlated nature of the aperiodic order. In addition, our results show that the Sinai transport exponent *v* can be largely controlled by the refractive index contrast. According to the microscopic Sinai transport theory the parameter *v* describes the degree of spatial non-homogeneity of transition rates (or scattering potentials) in the system. Our data demonstrates that this exponent can be tailored over a large range in the case of photon scattering, as shown in Fig. 5(c). In the inset of Fig. 5(c) we also display the calculated ACF decay curves for different values of refractive index contrast, vertically translated for better visualization. Notice that for structures with multifractal spectra it is not possible to associate a unique scaling exponent to the ACF decay. However, our data demonstrate an approximate inverse power-law scaling of the ACF of the RS structure. This behavior can be attributed to the contribution of the leading dimension in the multifractal spectrum, shown in the methods section. However, it is presently not possible to establish a simple relation between the ACS decay and the multifractal transmission spectrum. We also observe that, differently from Fibonacci and Thue-Morse structures, the ACF decay curves of RS structures present a weaker oscillatory behavior. This is consistent with the more inhomogeneous character of multifractal systems^26^ with a very broad singularity spectrum (shown in the methods section). However, to what extent Sinai logarithmic sub-diffusion of light is a generic attribute of wave propagation in multifractal aperiodic media remains to be investigated in the future.

Finally it is interesting to realize that in anomalous sub-diffusive processes the diffusion constant vanishes asymptotically, i.e., $D={\mathrm{lim}}_{t\to \infty }\frac{\langle {x}^{2}(t)\rangle }{t}$. Therefore, photon sub-diffusion provides a mechanism to inhibit diffusive transport that fundamentally differs from traditional Anderson localization in random media. Moreover, it is important to notice that Sinai-type sub-diffusion occurs under conditions such that the localization length of the modes in the RS structure is larger than the system size, implying that interference effects are very weak. This situation is in stark contrast with Anderson localization in random media that requires strong wave interference effects. In order to demonstrate this important feature of Sinai sub-diffusion we performed analytical and numerical calculations of the localization length and the scattering mean free path in the RS structure as a function of refractive index contrast (see methods section). The results clearly show that Sinai sub-diffusion occurs for values of the refractive index contrast such that the localization length exceeds the size of the RS system, i.e., outside the wave localization range. Therefore, the demonstration of logarithmic sub-diffusion in RS multifractal systems provides novel opportunities to inhibit the diffusion of optical waves in deterministic dielectric environments at relatively small values of the refractive index contrast. In the emerging technological opportunities offered by aperiodic structures in photonic metamaterials^11, 48, 49^, the engineering of deterministic systems with logarithmic-in-time processes can directly impact optical devices due to the enhancement of the photon residence time (or dwelling time) intrinsically associated to sub-diffusion processes. By increasing light-matter coupling in a scattering medium over what is possible using classical transport mechanisms, logarithmic sub-diffusion paves the way to broadband photonic trapping and localization effects even in the weak scattering regime, where a modified (fractional) diffusion picture can be applied. Since such mechanisms fundamentally do not rely on strong wave interference effects the resulting photon transport is broadband, naturally stimulating novel applications to active photonic devices such as light sources, absorbers, and optical sensors.

## Conclusions

In conclusion, we systematically investigated the propagation of optical wave packets through primary examples of periodic, quasi-periodic and pseudo-random photonic multilayer structures and we demonstrated largely tunable anomalous photon transport, including logarithmic Sinai sub-diffusion of photons for the first time. In particular, while optical pulses in self-similar Fibonacci and Thue-Morse systems with monofractal energy spectra obey power-law anomalous scaling, in pseudo-random RS structures with multifractal energy spectra they are transported logarithmically, in agreement with the analytical predictions of a distributed-order fractional diffusion model. The fruitful connection between fractional transport equations and photon transport in deterministic aperiodic media has been established providing novel insights into the complex behavior of multiple light scattering in non-homogeneous environments with tunable spatial correlations. The demonstration of novel photon transport regimes including logarithmic sub-diffusion in deterministic optical media provides novel degrees of freedom to tailor light-matter interactions and to engineer unusual pulse propagation and wave dispersion phenomena in optical devices. This is in stark contrast with the traditional vision that predicts the vanishing of the diffusion constant only in the strong scattering regime due to the interplay of multiple interference and disorder effects. In our paper we demonstrated an alternative path to localization that relies on a different transport mechanism other than strong scattering. This feature can be very advantageous in many device contexts where strongly scattering (i.e., high refractive index) materials are not readily available or where broad band frequency responses are desirable. Moreover, since materials with large refractive index and with minimal absorption across broad frequency spectra are not readily available, the engineered photon sub-diffusion approach may provide a different route to wave localization that relaxes such stringent materials requirements. We envision that our demonstration of tunable sub-diffusion of optical waves in deterministic media will enable the development of novel thin-film light trapping devices such as solar cells and photodetectors where light absorption efficiency can be significantly enhanced by slowing down the photon transport in weakly absorbing optical media. Finally, engineered sub-diffusion of photons may lead to a fundamentally novel strategy to boost light-matter interaction in low-index, structurally complex aperiodic laser structures where, similarly to the case of random lasers, the lasing threshold relates directly to the photon diffusion constant.

## Methods

### Electromagnetic calculations

The calculation of the electromagnetic fields through multilayer structures has been performed using the scattering matrix method and the electromagnetic pulse transport in time-domain obtained by Fourier synthesis. The central frequency of the pulse is ${\omega }_{0}=2\pi c/{\lambda }_{0}$, where *λ*_0_=1550 *nm*. The incident field *E*_*i*_(*x* = 0) is described by a Gaussian spectral profile as: ${E}_{i}(x=\mathrm{0,}\,\omega )={E}_{0}exp(\,-\,{a}^{2}{(\omega -{\omega }_{0})}^{2})$, with *E*_0_ = 1 and *a* = 33 *fs*. The propagation of waves occurs normally to the layers (i.e., along *x* axis) and the layers are homogeneous in the *yz*-plane. The electric field is polarized along *y* axis. Under these conditions, the electric field at a given position *x* within the sample can be expressed as:

6 $$E(x,t)={\int }_{0}^{\infty }{E}_{i}\mathrm{(0,}\,\omega ){e}^{-i\omega t}\psi (x,\omega )d\omega $$

where $\psi (x,\omega )$ is the so-called scattering map^35^.

For an arbitrary layered structure, *ψ*(*x*, *ω*) can be expressed in closed form as:

7 $$\psi (x,\omega )=[\prod _{m=1}^{j-1}{t}_{m}(\omega )]\,[{e}^{ik{n}_{j}(x-{x}_{j-1})}+{r}_{j}(\omega ){e}^{-ik{n}_{j}(x-{x}_{j})}]={\rm{\Phi }}(x,\omega )\chi (x,\omega )$$

where ${x}_{j-1} < x < {x}_{j}$ are inside the *j* ^*th*^ layer in the structure, *k* is the free space wavenumber and *n*_*j*_ represents the refractive index of *j* ^*th*^ layer. The reflection and transmission functions *r*_*j*_(*ω*) and *t*_*j*_(*ω*) are obtained iteratively for each layer using the recursive relations:

8 $${r}_{j}(\omega )=\frac{({n}_{j+1}-{n}_{j}){\varphi }_{j}+2{n}_{j}{\varphi }_{j+1}{r}_{j+1}(\omega ){t}_{j+1}(\omega )}{{n}_{j+1}+{n}_{j}}$$

9 $${t}_{j}(\omega )=\frac{2{n}_{j}{\varphi }_{j-1}}{({n}_{j-1}+{n}_{j})+({n}_{j-1}-{n}_{j}){\varphi }_{j}{r}_{j}(\omega )}$$

assuming *r*_*N*_ = 0 and *t*_*N*_ = 1, where N represents the total number of layers and ${\varphi }_{j}=exp(ik{n}_{j}({x}_{j}-{x}_{j-1}))$ is the propagation phase inside each layer. Equations (8) and (9) represent a compact form of scattering matrix equations for waves propagating at normal incidence to the layers^50^.

### Heuristic scaling analysis

**Figure 6:**
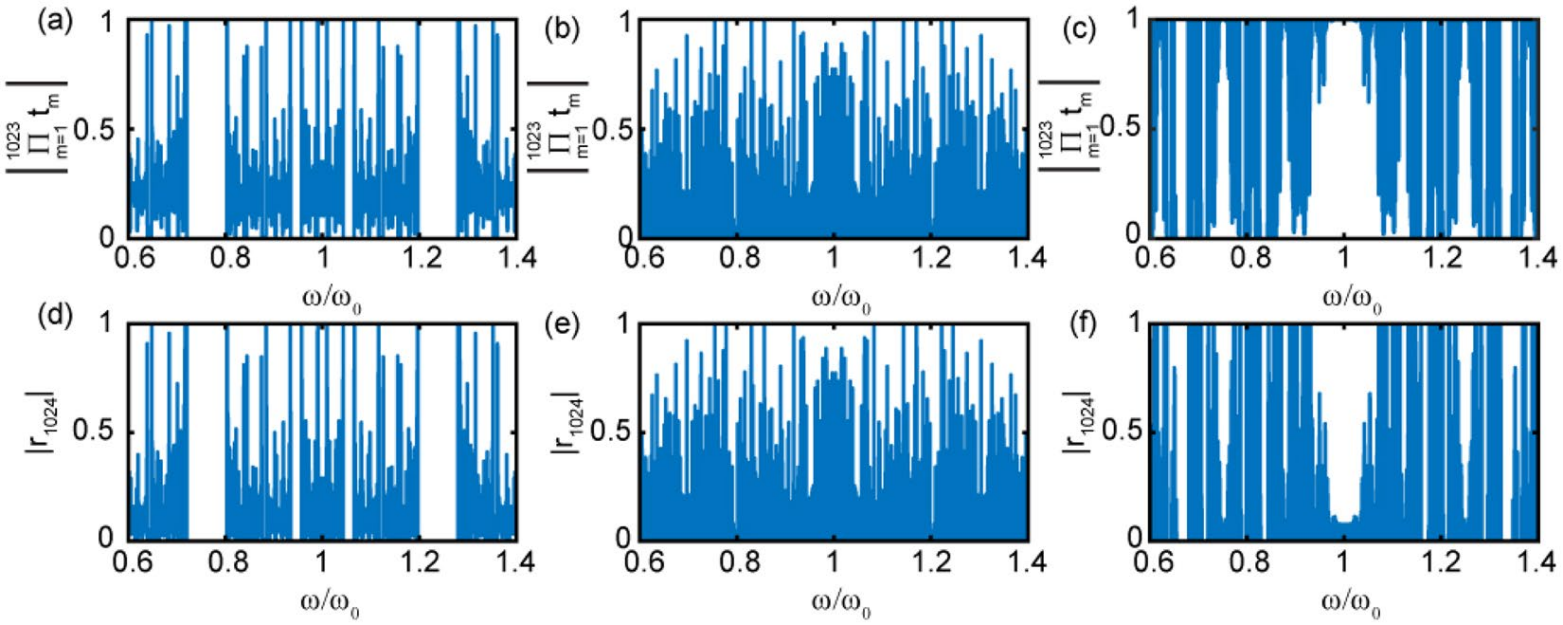
Net transmission (**a–c**) and reflection (**d–f**) spectrum defined using equations (8) and (9) respectively in the middle layer of the samples investigated in the main text. Each structure has 2048 layers and the panels display the net reflection and transmission values at the 1024^*th*^ layer. (**a**,**d**) Correspond to Fibonacci multilayers with *n*_1_ = 1.5 and *n*_2_ = 1.9. (**b**,**e**) Correspond to Rudin-Shapiro multilayers with *n*_1_ = 1.5 and *n*_2_ = 1.75 and (**c**), (**f**) correspond to the Thue-Morse multilayers with *n*_1_ = 1.5 and *n*_2_ = 1.75.

The scattering map *ψ*(*x*, *ω*) of aperiodic structures with fractal spectra is a self-affine two-dimensional function of space and frequency in the limit of large sample size *L*. A self-affine function describes fractality with different scale-invariant symmetries along the *x*- and *ω*-directions. Indeed we show in Fig. (6) that, for a large sample length *L*, *ψ*(*x*, *ω*) approaches the fractal transmission spectrum of the corresponding aperiodic structure. More precisely, it has been shown that the counting function, or the integrated density of states, of a fractal spectrum centered around the central frequency *ω*_0_ follows the power-law scaling relation: ${{\rm{\Gamma }}}_{{\omega }_{0}}(\omega )={|\omega -{\omega }_{0}|}^{\beta }G(\mathrm{ln}|\omega -{\omega }_{0}|/\gamma )$ where *G* is a log-periodic function, *β* and *γ* are scaling constants that depend on the choice of the central frequency^51^. In addition, both the *r*_*j*_(*ω*) and the product of the *t*_*j*_(*ω*) are self-similar functions, as illustrated in Fig. (6). For a fixed value of *ω*, the function *ψ*(*x*, *ω*) provides the spatial profile of the corresponding critical mode. In general, critical modes are non-uniform fractals, or multifractal signals, characterized by a distribution of scaling exponents that depends on the choice of the optical frequency *ω*. Therefore, in the large *L*-value limit the *ψ*(*x*, *ω*) is asymptotically self-affine with local scale-invariance symmetry: $\psi (ax,b\omega )={a}^{\alpha }{b}^{\beta }\psi (x,\omega )$ for *α* and *β* positive real numbers that depend in general on the local position in space and frequency.

In order to justify the anomalous scaling of the transport in the considered aperiodic structures we need to address the time evolution of the optical pulses. This can be done by considering the the scattering map in the space-time domain *ψ*(*x*, *t*), which is obtained by inverse Fourier transforming Equation (7):

10 $$\psi (x,t)=\tilde{{\rm{\Phi }}}(x,t)\otimes \tilde{\chi }(x,t)$$

where $\tilde{{\rm{\Phi }}}(x,t)$ and $\tilde{\chi }(x,t)$ are the inverse Fourier transforms of the functions ${\rm{\Phi }}(x,\omega )$ and $\chi (x,\omega )$ defined in (7).

**Figure 7:**
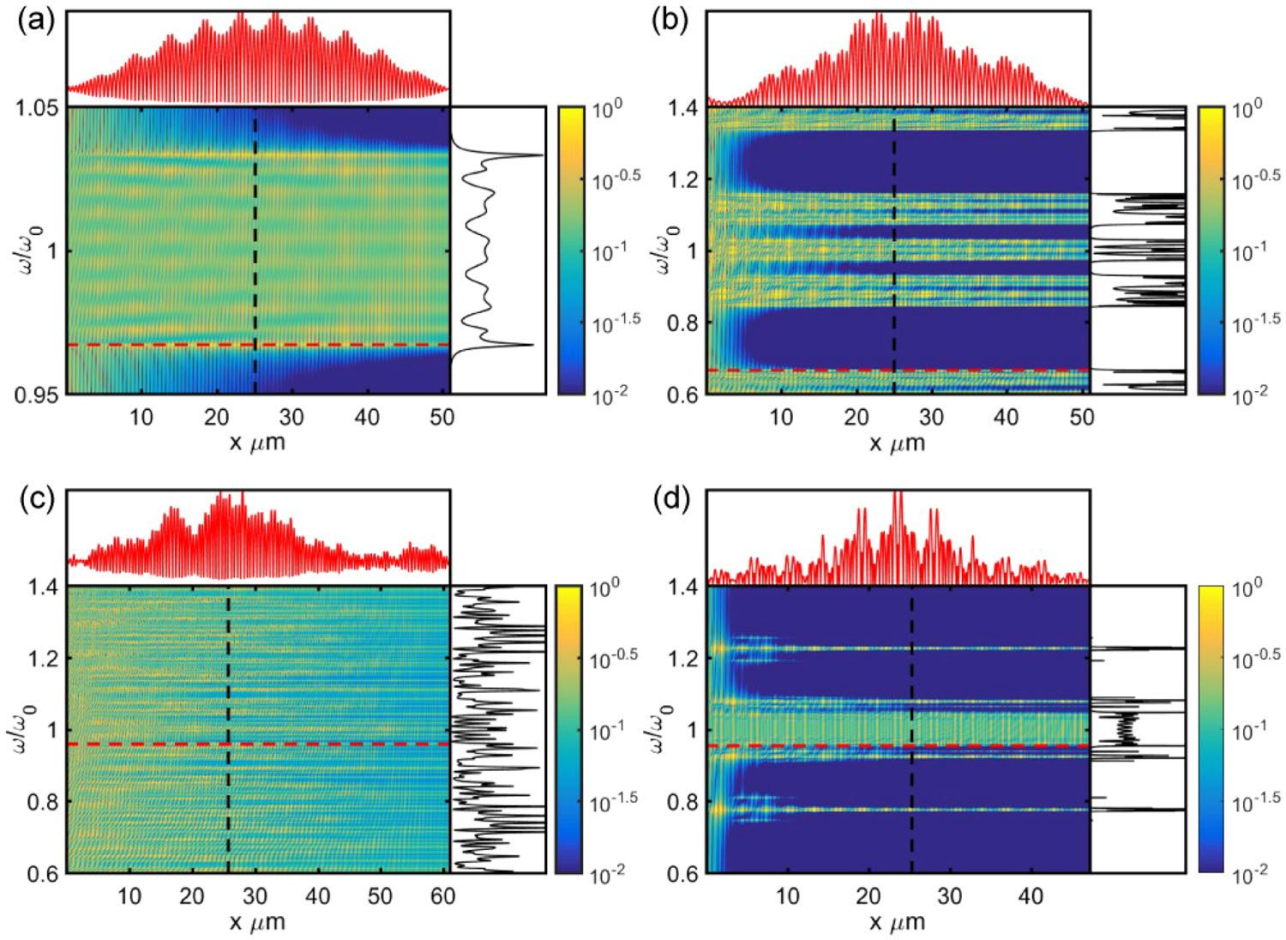
Scattering Map of fields in different sequence of layers. (**a**) Periodic micro cavities (*n*_1_=1.5 and *n*_2_ = 1.65; 207 Layers), (**b**) Fibonacci (*n*_1_ = 1.5 and *n*_2_ = 2.5; 233 Layers), (**c**) Rudin-Shapiro (*n*_1_ = 1.5 and *n*_2_ = 1.7; 256 Layers) and (**d**) Thue-Morse (*n*_1_ = 1.5 and *n*_2_ = 3.5; 256 Layers). Color represents $|\psi (x,\omega )|$ calculated using equation (7).

**Figure 8:**
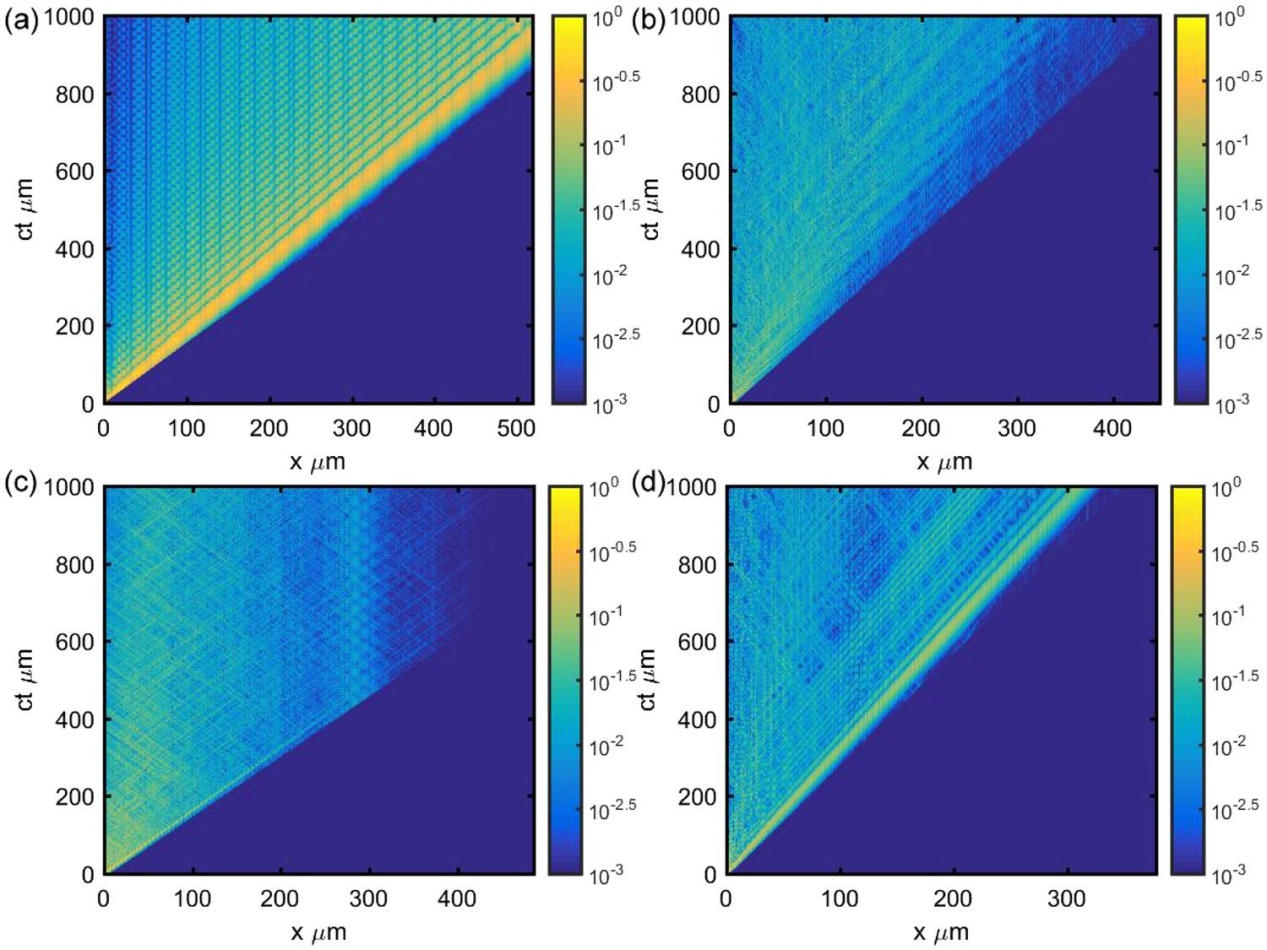
Time evolution of optical pulse in different sequence of layers. (**a**) Periodic micro cavities (*n*_1_ = 1.5 and *n*_2_ = 1.65; 207 Layers), (**b**) Fibonacci (*n*_1_ = 1.5 and *n*_2_=2.5; 233 Layers), (**c**) Rudin-Shapiro (*n*_1_ = 1.5 and *n*_2_=1.7; 256 Layers) and (**d**) Thue-Morse (*n*_1_ = 1.5 and *n*_2_=3.5; 256 Layers). Color represents $|\psi (x,t)|$ calculated using equation (10). ‘ct’ in all panels is the time equivalent length where c represents the velocity of light in vacuum and t represents time.

Despite the non-analytic and complex character of the functions involved, Equation (10) defines a non-local convolution that reveals the long-range correlated nature of the wave scattering in layered systems. Assuming a local scaling relation $\psi (x,\omega )\sim C{x}^{\alpha }{\omega }^{\beta }$ and remembering that power law functions are invariant under both Fourier transformation and convolution operations, it follows immediately that *ψ*(*x*, *t*) is asymptotically self-affine as well. The computed scattering maps *ψ*(*x*, *ω*) and *ψ*(*x*, *t*) for the investigated aperiodic photonic structures are shown in Figs (7) and (8) along with the electric field distributions of few representative critical modes.

### Connection with anomalous transport

The above scaling analysis allows us to establish that both the ACF and the MSD of photonic structures characterized by self-affine scattering maps exhibit anomalous transport behavior according to a nonlinear (power-law) scaling with respect to time. In particular, by noticing that the ACF is the time integral of the scattering map, and that any causal power function of the form ${{\rm{\Phi }}}_{\mu }(t)={t}^{\mu -1}/{\rm{\Gamma }}(\mu )$ transforms into ${{\rm{\Phi }}}_{\mu +1}(t)$ when integrated between zero and *t*, it follows that ACF must exhibit power-law scaling asymptotically, consistently with our numerical simulations. A similar argument can be made to support the power-law scaling of the MSD as well. On the other hand, in structures with multifractal transmission spectra, as the RS systems discussed in the main text, it is not possible to asymptotically represent the scattering maps *ψ*(*x*, *ω*) and *ψ*(*x*, *t*) as power-laws due to the broad distributions of their scaling exponents. As explained in the text, when the weight function is a power-law, then ultra-slow Sinai sub-diffusion arises in the MSD scaling. No closed-form results are known for the corresponding ACF decay in this case.

### Connection with fractional dynamics

In the previous section we have shown that both the space-frequency *ψ*(*x*, *ω*) and the space-time *ψ*(*x*, *t*) scattering maps of fractal structures are self-affine objects that can be expressed asymptotically as a convolution integral with a power-law kernel. This feature is the hallmark of fractional calculus since fractional operators are defined by convolution integrals with non-local power-law kernel functions^16^. The fractional (non-local) character of the time-dynamics of optical transport across fractal structures is exemplified by the scattering maps *ψ*(*x*, *t*) plotted in Fig. 8. The non-Markovian (i.e., correlated) nature of the pulse dynamics is clearly evidenced by the fractal distributions of internal pulse reflections that characterize aperiodic systems (Fig. 8b–d).

### Solutions of the FDWE

The fundamental solutions (i.e., Green’s functions) corresponding to the Cauchy problems of the FWDE in Equation (3) are denoted by ${G}_{\beta }^{(j)}(x,t)$ (*j* = 1, 2) and can be analytically obtained^34^. In particular, by introducing the similarity variable $x/{t}^{\beta \mathrm{/2}}$ we can express the two Green’s functions in terms of the one-variable *reduced Green’s functions* ${K}_{\beta }^{(j)}(x)$ (*j* = 1, 2) as follows:

11 $${G}_{\beta }^{\mathrm{(1)}}(x,t)={t}^{-\beta \mathrm{/2}}{K}_{\beta }^{\mathrm{(1)}}(x/{t}^{\beta \mathrm{/2}})$$

12 $${G}_{\beta }^{\mathrm{(2)}}(x,t)={t}^{-\beta \mathrm{/2}+1}{K}_{\beta }^{\mathrm{(2)}}(x/{t}^{\beta \mathrm{/2}})$$

A convenient expression for the Green’s functions as convergent power series has been recently derived^34^:

13 $${K}_{\beta }^{(j)}(x)=\frac{1}{2}\sum _{n=0}^{{\rm{\infty }}}\frac{{(-x)}^{n}}{n!{\rm{\Gamma }}[-\beta n/2+(j-\beta /2)]}\,;{\rm{j}}=1,2$$

Both the Green functions above have been shown to be non-negative and normalized, so they can be interpreted as probability density functions.

### Localization length analysis

**Figure 9:**
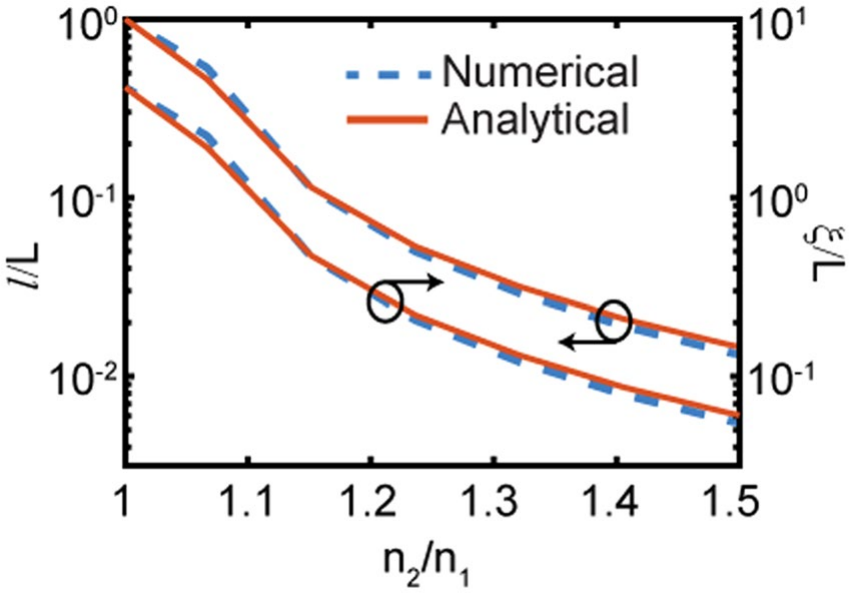
Localization length and scattering mean free path of the RS sample as a function of refractive index contrast. Dashed lines represent localization length normalized to the sample length. Solid lines represent the same calculated using the analytical formula derived for random systems^53^.

In Fig. 9 we estimate the localization length *ξ* and photon mean free path *l* for RS structures as a function refractive index contrast. We also compare the results with the case of binary pseudo-random structures with the same total length. The data in Fig. 9 demonstrate excellent agreement between the analytical theory valid for 1D random structures^52, 53^ and the numerical results on RS structures obtained from the scaling of their transmission spectra. These results indicate that the scattering mean free path is larger than the total length *L* of the samples for the range of refractive index contrast where Sinai sub-diffusion is reported. This implies that logarithmic photon transport is strongly influenced by spatial correlations in wave interference, which develop even for small values of the refractive index contrast if *L* is sufficiently large.

The localization length of pseudo-random and RS structures is calculated using the ensemble-averaged transmission over the frequency spectrum and it is defined by ref. 52: $\xi /L=-\,\mathrm{1/}\langle \mathrm{ln}\,T\rangle$, where *T* is the transmission coefficient of the sample and *L* is the total length of the sample. To calculate the localization length in 1D random multilayers we averaged over the frequency spectrum of the optical pulse the analytical expression provided in ref. 53:

14 $$\xi (\lambda )=\mathrm{2(}{d}_{A}+{d}_{B}){\{\mathrm{ln}[(3{n}_{A}^{2}+{n}_{B}^{2})({n}_{A}^{2}+3{n}_{B}^{2})+\frac{3{({n}_{A}^{2}-{n}_{B}^{2})}^{2}}{4\cos (\pi {\lambda }_{0}/\lambda )-5}]-2\mathrm{ln}\mathrm{(4}{n}_{A}{n}_{B})\}}^{-1}$$

The scattering mean free path *l* is estimated using the approximate expression valid for 1D random systems:

15 $$\xi \simeq \mathrm{(1}+\pi )l$$

### Wavelet Transform Modulus Maxima (WTMM) method

This powerful mathematical technique was introduced to investigate the hierarchical structure of singular signals and it is particularly suited to characterize fractal and multifractal measures with non-isolated singularities such as the spectra of the aperiodic structures discussed in the main text.

The method enables the computation of the singularity spectrum *D*(*α*) of a multifractal signal or measure by analyzing the scaling properties of a global partition function defined on the maxima of the modulus of the wavelet transform of the signal. The partition function is defined as:

16 $$Z(q,s)=\sum _{n}{|Wf({u}_{n},s)|}^{q}$$

where $|Wf({u}_{n},s)|$ is the modulus of the wavelet transform of the function *f* and *u*_*n*_ (*n* integer) denotes the position of the local maxima of *f* at a given scale *s* and *q* is a real number. For each value of *q*, the partition function features a power-law scaling according to:

17 $$Z(q,s)\sim {s}^{\tau (q)}$$

at fine scales. All maxima that do not propagate up to the finest scales are typically removed in the calculation of the partition function. The singularity spectrum of the multifractal can now be obtained by computing the Legendre transform of *τ*(*q*):

18 $$D(\alpha )=\mathop{\min }\limits_{q}[q(\alpha +\mathrm{1/2)}-\tau (q)]$$

Additional details on wavelets and multifractal analysis can be found in ref. 54.

**Figure 10:**
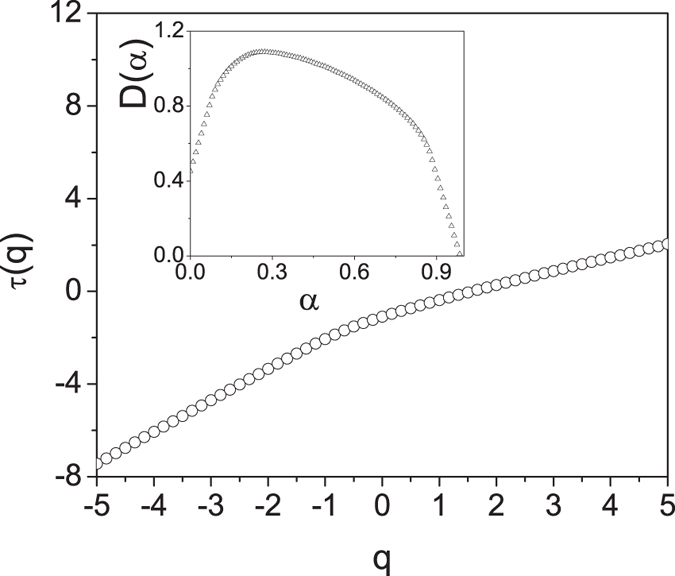
Scaling exponent *τ*(*q*) of the optical transmission of a Rudin-Shapiro structure (1024 layers). The inset shows the corresponding singularity spectrum $D(\alpha )$ calculated numerically with a Legendre transform of *τ*(*q*). The wavelet transform $WT(u,s)$ of the optical transmission was computed using an analyzing wavelet *ψ* = −*θ*′′ where *θ* is a Gaussian wavelet.

We prove the multifractality of the RS transmission spectrum by computing its singularity spectrum using the free library of MATLAB wavelet routines WaveLab850^55^. The non-linear behavior of the *τ*(*q*) exponent in Fig. (10) demonstrates the multifractal nature of the signal. Moreover, besides a nonlinear *τ*(*q*) exponent, multifractals are also characterized by a distinctive single-humped (concave) shape spectrum *D*(*α*)^41^. The singularity spectrum of the RS transmission spectrum is shown in the inset of Fig. (10). Indeed, a very broad singularity spectrum is obtained consistently with the multifractality of the RS structure. In contrast, mono-fractals spectra, such as Fibonacci quasi-periodic structures, feature a linear scaling exponent *τ*(*q*) and the *D*(*α*) singularity spectrum is supported by a single point coinciding with their fractal dimension.
